# Precision Nutrition in Type 2 Diabetes Prevention Through Molecular Nutrigenomic and Epigenetic Modulation of Insulin Signaling and Glucose Metabolism

**DOI:** 10.3390/ijms27041631

**Published:** 2026-02-07

**Authors:** Daniel Rumui, Aida Dama, Era Gorica, Victor Samuel Halim, Apple Faith Setiawan, Xandra Christensen Tjia, Edwin Hadinata, Dante Saksono Harbuwono, Fahrul Nurkolis, Antonello Santini

**Affiliations:** 1 School of Medicine, Faculty of Medicine, Ciputra University of Surabaya, Surabaya 60219, Indonesia; 2 Department of Pharmacy, Faculty of Medical Sciences, Albanian University, 1017 Tirana, Albania; 3 Department of Cardiac Surgery, Faculty of Medicine, University Hospital Zürich, University of Zürich, Wagistrasse 12, 8952 Schlieren, Switzerland; 4 Division of Endocrinology, Metabolism, and Diabetes, Department of Internal Medicine, Faculty of Medicine, Universitas Indonesia, Dr. Cipto Mangunkusumo National Referral Hospital, Jakarta 10430, Indonesia; 5 Master of Basic Medical Science, Faculty of Medicine, Universitas Airlangga, Surabaya 60131, Indonesia; 6 Medical Research Center of Indonesia, Surabaya 60281, Indonesia; 7 Institute for Research and Community Service, State Islamic University of Sunan Kalijaga (UIN Sunan Kalijaga), Yogyakarta 55281, Indonesia; 8 Department of Pharmacy, University of Napoli Federico II, Via Domenico Montesano, 49, 80131 Napoli, Italy

**Keywords:** precision nutrition, nutrigenomics, epigenetics, insulin signaling, glucose metabolism, type 2 diabetes prevention, gene–diet interaction, DNA methylation, metabolic inflammation, functional foods

## Abstract

Precision nutrition has emerged as a promising strategy for the prevention of type 2 diabetes mellitus (T2DM) by targeting molecular pathways underlying insulin resistance and impaired glucose metabolism. Accumulating evidence indicates that dietary patterns, caloric intake, and specific nutrients can modulate gene expression and epigenetic mechanisms involved in insulin signaling, inflammation, and energy homeostasis. This narrative review synthesizes recent human and experimental studies (2025–2026) examining how dietary components influence transcriptional and epigenetic regulation of insulin signaling and glucose metabolism in the context of T2DM prevention. A total of 29 peer-reviewed studies were included, encompassing dietary patterns, macronutrient manipulation, micronutrient and bioactive supplementation, and gene–diet interactions. Very-low-calorie diets consistently induced coordinated modulation of key metabolic genes, including downregulation of *glucose transporter type 4* (*GLUT4*) and upregulation of *PDK4*, *CPT1*, and *AMPK*, reflecting a metabolic shift toward enhanced fatty acid oxidation and improved insulin sensitivity. In contrast, high-fat and fructose-rich diets promoted proinflammatory gene expression and immune activation, contributing to insulin resistance. Plant-based and vegan dietary patterns were associated with reduced epigenetic aging and improved insulin sensitivity through DNA methylation changes. Targeted interventions, including vitamin D combined with probiotics, dietary fiber, nucleotides, and trace elements such as copper, further demonstrated favorable transcriptional and epigenetic effects linked to improved glycemic control. Collectively, these findings highlight diet-driven modulation of insulin signaling and glucose metabolism at the molecular level and support nutrigenomics-guided precision nutrition as a viable preventive approach for T2DM. Integrating genetic and epigenetic insights into dietary strategies may enable more personalized and effective interventions to curb the growing global burden of type 2 diabetes.

## 1. Introduction

Conventional dietary recommendations for type 2 diabetes mellitus (T2DM) prevention remain largely population-based, emphasizing macronutrient balance, caloric restriction, and lifestyle modification [1]. However, accumulating evidence indicates that interindividual variability in genetic architecture, epigenetic regulation, and metabolic responsiveness profoundly influences dietary efficacy [2]. This has led to the emergence of precision nutrition, an approach that integrates nutrigenomics, epigenetics, and metabolic phenotyping to tailor dietary strategies according to molecular responses rather than generalized guidelines.

T2DM arises due to insulin resistance, where cells are unable to effectively utilize insulin, either with or without absolute insulin deficiency [3,4]. Type 2 diabetes constitutes the predominant form, representing more than 85% of the total diabetes burden globally [5,6]. The condition is fundamentally defined by hyperglycemia, which occurs when the body cannot effectively utilize the insulin it produces [7]. This metabolic derangement affects not only carbohydrate metabolism but also protein and fat metabolism, leading to widespread physiological consequences [8]. Diabetes represents one of the most significant global health challenges of modern times, contributing substantially to mortality, morbidity, and healthcare expenditure across all nations [9,10].

The worldwide prevalence of diabetes has reached epidemic proportions, with approximately 536.6 million adults aged 20–79 years living with diabetes in 2021, representing 10.5% of the global population [10,11]. This burden is projected to increase dramatically, with prevalence expected to reach 578 million by 2030 and 700 million by 2045 [12]. The vast majority of individuals with diabetes reside in low- and middle-income countries, where access to care and insulin remains challenging. The economic impact is substantial, with global healthcare expenditure on diabetes expected to reach $760 billion [9,13].

The fundamental pathophysiology of type 2 diabetes involves two primary factors: defective insulin secretion by pancreatic β-cells and the inability of insulin-sensitive tissues to respond appropriately to insulin [14,15]. In T2DM, the lack of β-cell compensatory mechanisms to overcome peripherally developed insulin resistance is a paramount factor leading to disturbed blood glucose levels and lipid metabolism [16]. This dual dysfunction is characterized by pancreatic insulin resistance and β-cell dysfunction [17].

Hyperglycemia is the main metabolic feature of T2DM because of insulin resistance and β-cell dysfunction [18]. Insulin release and activity are essential processes for glucose homeostasis. The molecular mechanisms involved in the synthesis and release of insulin, as well as in its detection, are tightly regulated. Defects in any of the mechanisms involved in these processes can lead to a metabolic imbalance responsible for the development of the disease [14]. Insulin binds to the insulin receptor (IR) on the cell surface, which causes the β-subunits to undergo autophosphorylation, recruiting IRS1 and IRS2 [19,20]. IRS activates PI3K, which activates AKT, also known as the PI3K/AKT pathway. These pathways ultimately promote GLUT4 exocytosis, allowing glucose to be transported into the cell [19].

Importantly, insulin signaling is not only regulated by endocrine factors but is highly sensitive to dietary cues at the transcriptional and epigenetic levels [21]. Nutrients, dietary patterns, and bioactive food components can modulate insulin receptor signaling, glucose transport, mitochondrial metabolism, and inflammatory tone through gene expression changes, DNA methylation, histone modifications, and non-coding RNAs [22]. These nutrigenomic and epigenetic mechanisms provide a molecular basis through which diet can actively reprogram metabolic health.

Multiple modifiable and non-modifiable risk factors contribute to diabetes development. Modifiable risk factors include unhealthy diet, physical inactivity, excessive weight gain, smoking, and sedentary lifestyles [13]. Obesity represents a particularly significant cause of insulin resistance, linked to metabolic syndrome components such as central adiposity and elevated triglycerides [23]. Thus, this review aims to summarize the impact of dietary components on gene expression related to insulin signaling and glucose metabolism to reduce risks of T2DM. This review explores the molecular regulation of insulin signaling and glucose metabolism by diet, an aspect of type 2 diabetes prevention that remains relatively underexplored in existing literature.

Although several reviews have discussed dietary interventions, insulin resistance, or nutrigenomics independently, most fail to integrate gene–diet interactions and epigenetic regulation of insulin signaling within a prevention-focused precision nutrition framework. Moreover, recent high-resolution human studies (2025–2026) leveraging transcriptomics, epigenomics, and multi-omics approaches remain under-synthesized.

## 2. Methodology

The research question of this review is “How do dietary components modulate gene expression related to insulin signaling and glucose metabolism in the prevention of type 2 diabetes?”. The screening for relevant studies was set to a time frame of 2025–2026 and performed using the Boolean operator method with the following keywords: TITLE-ABS-KEY ((“food” OR “diet” OR “nutrition” OR “nutrient” OR “functional food” OR “bioactive compound”) AND (“nutrigenomics” OR “nutrigenetics” OR “gene expression” OR “epigenetic” OR “DNA methylation”) AND (“insulin sensitivity” OR “insulin resistance” OR “type 2 diabetes” OR “diabetes prevention”)). Through these keywords, 424 studies were identified, and were further filtered by the authors, resulting in 29 peer-reviewed articles.

## 3. Results

A total of twenty-nine (29) peer-reviewed articles were included for this review. All studies reported at least one molecular or gene expression outcome. The characteristics of the included studies are shown in Table 1.

Among the twenty-nine studies included, the most reported outcomes were changes in metabolic genes, pathways, or metabolic traits (seven studies). This was followed by inflammation or cytokine-related outcomes (six studies), epigenetic modifications (5 studies), genetic variants or single-nucleotide polymorphisms (five studies), and measures of insulin signaling, resistance, or sensitivity (five studies).

### 3.1. Macronutrient-Specific Effects on Gene Expression

**Figure 1:**
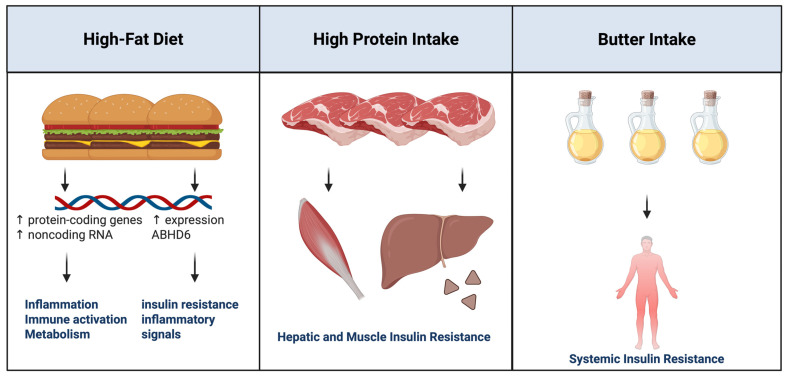
Macronutrient-specific effects on insulin resistance and gene expression. This figure was created by the author (Fahrul Nurkolis and Daniel Rumui) using licensed BioRender.com. Upward arrows (↑) denote an increase or activation, whereas downward arrows (↓) denote a decrease or inhibition of the indicated parameters.

High-fat diets and single high-fat meals were associated with changes in gene expression related to inflammation, immune activation, and metabolism (Figure 1). These responses included the upregulation of protein-coding genes as well as noncoding RNAs, such as microRNAs and long noncoding RNAs, in peripheral blood mononuclear cells and adipose tissue [39]. High-fat dietary intake also increased expression of alpha/beta hydrolase domain-containing protein 6 in visceral adipose tissue. Notably, knockout models for this gene demonstrated enhanced insulin sensitivity and reduced inflammatory signaling, suggesting a potential causal role in diet-induced metabolic dysfunction [41].

Postprandial protein loads were most strongly associated with hepatic and muscular insulin resistance, whereas butter intake was linked to systemic insulin resistance. These distinct physiological responses were accompanied by unique molecular signatures, indicating that different nutrient classes activate discrete regulatory pathways [40].

### 3.2. Effects of Dietary Patterns on Gene Expression

**Figure 2:**
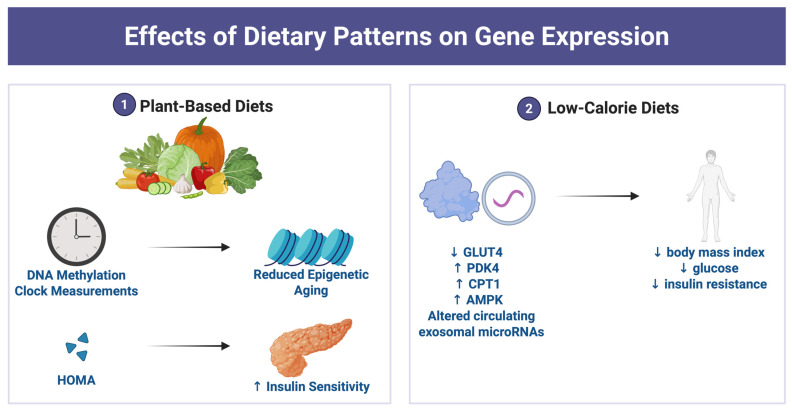
Effects of dietary patterns on gene expression. This figure was created by the author (Fahrul Nurkolis and Daniel Rumui) using licensed BioRender.com. Upward arrows (↑) denote an increase or activation, whereas downward arrows (↓) denote a decrease or inhibition of the indicated parameters.

Plant-based and vegan dietary patterns were associated with favorable molecular and metabolic profiles (Figure 2). Participants adhering to these diets exhibited reduced epigenetic aging, reflected in DNA methylation clock measures, along with improved insulin sensitivity. Abstaining from pork consumption was associated with lower biological age, while abstaining from poultry corresponded with higher biological age, suggesting differentiated epigenetic effects of specific animal-derived foods [33].

Low-calorie and weight-loss interventions also produced significant metabolic and molecular adaptations. Very-low-calorie diets (800–835 kcal/day) and structured weight-loss programs resulted in marked reductions in body mass index, glucose concentrations, and insulin resistance [35,51]. These physiological changes were accompanied by coordinated modulation of gene expression, including downregulation of glucose transporter type 4 (GLUT4) and upregulation of pyruvate dehydrogenase kinase 4 (PDK4), carnitine palmitoyltransferase 1 (CPT1), and AMP-activated protein kinase (AMPK) [35]. Additionally, similar weight-loss interventions related to diet restriction altered circulating exosomal microRNAs associated with type 2 diabetes remission, underscoring the responsiveness of regulatory networks to energy restriction [51].

### 3.3. Individual Nutrient Effects

Eight (8) studies reported the effects of various dietary interventions on gene expression, epigenetic regulation, and metabolic outcomes related to insulin sensitivity and metabolic health. Table 2 shows the dietary interventions and their effects on gene expression and metabolic effects.

Three studies reported upregulation of genes or signaling pathways, including increased expression of PDK4, CPT1, and AMPK following a very-low-calorie diet, activation of GPR120 and PPARγ in response to polyunsaturated fatty acids, and increased TLR2 expression and proinflammatory cytokine signaling associated with fructose intake [28,35,44].

In contrast, three studies observed downregulation of gene expression or related metabolic markers, including reduced GLUT4 expression following a very-low-calorie diet, decreased TNF-α activity with vitamin D plus probiotic supplementation, and reduced insulin levels and HOMA-IR following increased dietary fiber intake [26,35,49]. Two studies reported epigenetic modifications, with nucleotide supplementation associated with a reduction in DNA methylation age and increased serum copper levels linked to decreased methylation at specific CpG sites [31,49].

Relating to the metabolic effects, four studies reported improvements in glycemic control or insulin resistance, including interventions involving a very-low-calorie diet, dietary fiber, nucleotide supplementation, and naringenin or naringenin-reduced graphene oxide [24,35,36,49]. Two studies demonstrated reductions in inflammatory markers, specifically with vitamin D plus probiotic supplementation and naringenin-based interventions [24,26]. Improvements in adiposity or lipid metabolism were observed in two studies, namely those examining naringenin-related compounds and polyunsaturated fatty acids [24,28]. One study identified a reduced risk of type 2 diabetes and cardiovascular disease associated with copper-related methylation changes [31]. Conversely, fructose intake was associated with a proinflammatory metabolic response [44].

### 3.4. Insulin Signaling Pathway Genes

**Figure 3:**
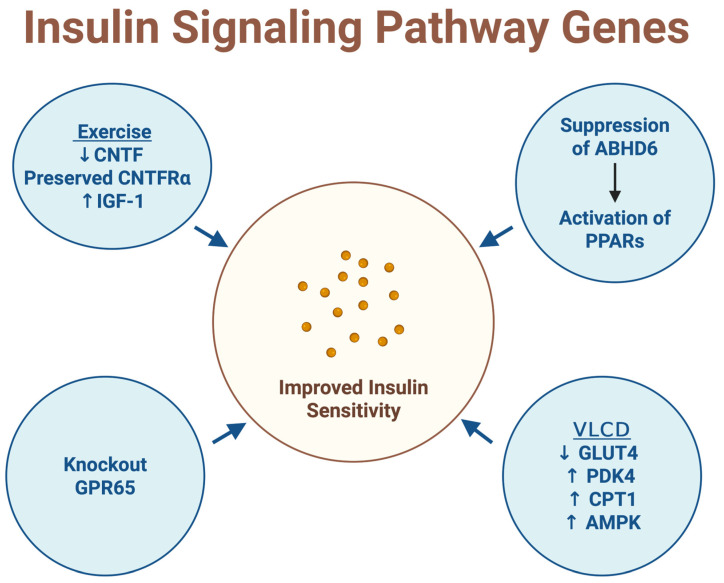
Insulin signaling pathway genes. This figure was created by the author (Fahrul Nurkolis and Daniel Rumui) using licensed BioRender.com. Upward arrows (↑) denote an increase or activation, whereas downward arrows (↓) denote a decrease or inhibition of the indicated parameters.

A very-low-calorie diet (VLCD) was shown to modulate insulin and metabolic genes, including the downregulation of GLUT4, alongside the upregulation of PDK4, CPT1, and AMPK (Figure 3). These coordinated changes were associated with improved insulin sensitivity, suggesting a metabolic shift toward enhanced fatty acid oxidation and energy sensing [35].

Exercise and dietary interventions in older adults also influenced insulin-related growth and neurotrophic signaling. Modulation of ciliary neurotrophic factor (CNTF), its receptor CNTFRα, and insulin-like growth factor 1 (IGF-1) was observed, with combined exercise interventions preserving CNTFRα expression and increasing IGF-1 levels. These changes were linked to improved insulin secretion and better physical function, highlighting the synergistic effects of exercise and diet on metabolic health during aging [29].

Adipocyte lipid metabolism and inflammation were influenced by alterations in ABHD6 and PPAR signaling. Suppression of alpha/beta hydrolase domain-containing protein 6 (ABHD6) in adipocytes increased intracellular monoacylglycerol levels, which in turn activated peroxisome proliferator-activated receptors (PPARs). This activation promoted anti-inflammatory and adipogenic gene programs, effectively decoupling obesity from insulin resistance [41].

Inflammatory and mitochondrial pathways were also implicated through GPR65. Knockout of G protein-coupled receptor 65 led to improved insulin sensitivity in diet-induced obese mice, accompanied by increased mitochondrial activity and reduced inflammation. These findings suggest a role for GPR65 in linking immune signaling, energy metabolism, and insulin resistance [52].

### 3.5. Glucose Metabolism Genes

**Figure 4:**
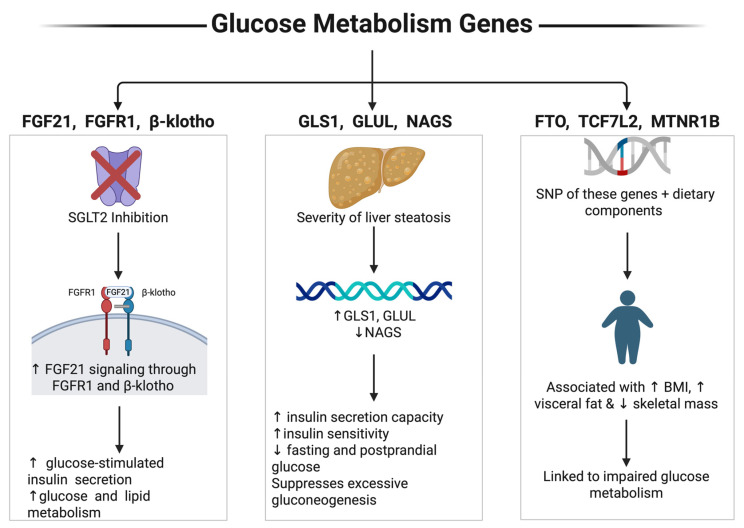
Glucose metabolism genes. This Figure was created by the author (Fahrul Nurkolis and Daniel Rumui) using licensed BioRender.com. Upward arrows (↑) denote an increase or activation, whereas downward arrows (↓) denote a decrease or inhibition of the indicated parameters.

Interventions targeting glucose handling and hormonal signaling affected the FGF21 axis (Figure 4). Sodium-glucose cotransporter 2 inhibition, in combination with fibroblast growth factor 21 (FGF21) signaling through FGFR1 and β-klotho, enhanced glucose-stimulated insulin secretion and improved both glucose and lipid metabolism. This highlights the therapeutic potential of modulating endocrine FGF signaling in metabolic disease [38].

Severity of steatotic liver disease was associated with changes in genes involved in glutamate and nitrogen metabolism, including GLS1, GLUL, and NAGS. The severity of steatotic liver disease correlated with altered expression of these genes, reflecting disruptions in hepatic amino acid metabolism and nitrogen handling that accompany progressive metabolic dysfunction [37].

Lastly, single-nucleotide polymorphisms in FTO, TCF7L2, and MTNR1B interacted with dietary factors such as protein intake, sugars, fiber, total energy, and saturated fatty acids. These gene–diet interactions modulated body mass index, insulin levels, and glycemic outcomes, underscoring the importance of personalized nutrition in metabolic health [25,36,40].

### 3.6. Epigenetic Modifications

Besides transcriptional profiles, dietary interventions also modulate epigenetics. Four (4) studies reported epigenetic modifications through dietary intervention, as shown in Table 3.

**Figure 5:**
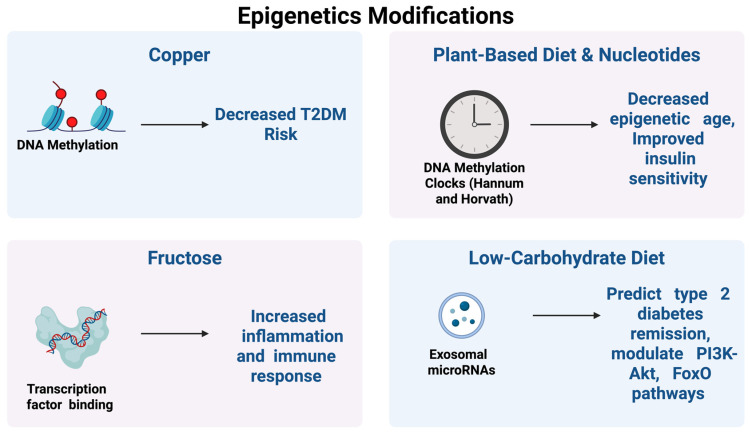
Epigenetic modifications by several nutrients. This Figure was created by the author (Fahrul Nurkolis and Daniel Rumui) using licensed BioRender.com. Upward arrows (↑) denote an increase or activation, whereas downward arrows (↓) denote a decrease or inhibition of the indicated parameters.

Copper intake is associated with changes in DNA methylation at specific CpG sites. Increased methylation at these sites was linked to a lower risk of type 2 diabetes and cardiovascular disease (Figure 5), suggesting that copper may influence metabolic health through epigenetic regulation [31]. Vegan dietary patterns, plant-based dietary patterns, and nucleotides affect DNA methylation clocks, which are composite measures (such as the Hannum and Horvath clocks) used to estimate biological or epigenetic age. The observed reduction in epigenetic age indicates a slowing of biological aging, and this was accompanied by improved insulin sensitivity, suggesting metabolic and longevity-related benefits of plant-based diets [33,49].

Low-carbohydrate diets were shown to alter exosomal microRNAs (including miR-92b-3p, miR-495-3p, and miR-452b-5p). These microRNAs modulate key insulin-signaling pathways such as PI3K-Akt and FoxO (Figure 5), and their expression patterns were able to predict remission of type 2 diabetes, indicating both mechanistic and potential biomarker relevance [51].

Fructose intake influences transcription factor binding, specifically involving TLR2 and SP1, which are important regulators of inflammatory and immune responses. Enhanced binding and activation of these transcription factors was associated with increased inflammation and immune activation, linking high fructose intake to adverse inflammatory outcomes [44].

## 4. Discussion

The evidence reviewed demonstrates that dietary patterns markedly influence gene expression and epigenetic regulation of insulin signaling and glucose metabolism. One of the most consistent findings across studies was the impact of energy restriction and VLCDs on insulin signaling genes. The downregulation of GLUT4 alongside upregulation of PDK4, CPT1, and AMPK suggests a metabolic shift from glucose utilization toward enhanced fatty acid oxidation and energy sensing. GLUT4 is a key mediator of insulin-stimulated glucose uptake, and reduced GLUT4 generally contributes to insulin resistance [53]. However, during energy restriction, lower GLUT4 expression may reflect adaptive metabolic reprogramming rather than impaired insulin sensitivity.

In contrast, high-fat and fructose-rich diets are consistently associated with insulin resistance. These findings are consistent with previous studies demonstrating a link between high-fat and high-fructose dietary patterns and the development of insulin resistance [54,55]. Evidence from our review suggests that insulin resistance induced by high-fat diets is mediated by chronic inflammation and impaired lipid handling in adipose tissue, processes driven in part by increased ABHD6 activity in obesity [41]. However, other studies have reported that insulin resistance may also result from the inhibition of key insulin-signaling genes, including IRS-2, PI3K, and AKT, indicating that insulin resistance arises through multiple interconnected mechanisms involving multiple gene expression alterations [54].

Plant-based and vegan dietary patterns have been associated with reduced epigenetic aging and improved insulin sensitivity, as evidenced by DNA methylation clock analyses. These findings extend prior epidemiological observations by offering a potential mechanistic explanation, whereby diets rich in dietary fiber, phytochemicals, and unsaturated fats may modulate DNA methylation at loci involved in insulin signaling and metabolic regulation [33]. Notably, specific CpG sites, including CPT1B and GNAS, have been linked to insulin sensitivity and glucose homeostasis [56]. Reduced epigenetic age has further been associated with lower cardiometabolic risk, suggesting that dietary interventions may influence not only metabolic health but also broader biological aging processes.

Nucleotide supplementation and trace element intake, particularly copper, were shown to modify DNA methylation at CpG sites associated with T2DM risk [31,49]. Imbalanced copper status (excess or deficiency) is known to impair insulin action and promote oxidative stress, but direct nutrigenomic evidence is sparse. Some animal work suggests that restoring copper in fructose-fed or diabetic models can rescue β-cell function and normalize metabolic genes, yet human data are limited [47].

Vitamin D and probiotic supplementation over 8 weeks decreased systemic inflammation and improved glycemic indices. Women receiving vitamin D (4000 IU/day) plus a Lactobacillus-containing probiotic showed significant drops in fasting insulin, HOMA-IR, and TNF-α gene activity relative to controls [26]. Similarly, other studies have shown that Vitamin D supplementation induces expression of glucose transporters and enzymes in insulin signaling (GLUTs, hexokinase, G6PC) and increases insulin receptor expression in muscle, adipose, and liver cells. Furthermore, vitamin D’s actions are partially epigenetic, as it has been shown that vitamin D enhances insulin receptor gene expression through epigenetic regulation [57]. In animal studies, probiotic supplements have been shown to reverse obesogenic epigenetic changes, indirectly modulating insulin pathways [58].

Despite promising mechanistic convergence, several limitations warrant consideration. First, heterogeneity in omics platforms (transcriptomics, methylomics, exosomal miRNA profiling) limits direct cross-study comparability and standardization. Second, many nutrigenomic findings remain context-dependent, influenced by baseline metabolic state, genetic background, tissue specificity, and intervention duration. Third, causal inference in human nutrigenomicsremains challenging, as many studies are observational or short-term interventions with limited longitudinal follow-up.

Translating molecular signatures into clinical precision nutrition strategies will require harmonized multi-omics pipelines, replication across populations, and integration with real-world dietary adherence data.

Looking ahead, the integration of AI-driven analytics, metabolomics, and personalized diet algorithms represents a critical next step for precision nutrition. Machine-learning models capable of integrating genomic variants, epigenetic markers, microbiome-derived metabolites, and dietary intake data may enable individualized prediction of insulin responsiveness to specific dietary patterns. Such approaches could shift diabetes prevention from generalized recommendations toward dynamic, molecularly informed nutritional prescriptions.

## 5. Recommendations

The findings of this review support several key recommendations. VLCD and structured weight-loss interventions may be considered effective short- to medium-term strategies when implemented under clinical supervision, as they have been shown to significantly reduce body mass index, glucose concentrations, and insulin resistance [35]. VLCDs should not be implemented on a daily basis. Instead, they may be more appropriately incorporated within a 5:2 intermittent fasting dietary pattern, in which very-low-calorie intake is restricted to two non-consecutive days per week. The 5:2 intermittent fasting approach has been shown to improve glycemic outcomes and promote short-term weight loss [59]. Other recommended dietary patterns include plant-based and vegan dietary patterns. These dietary patterns exhibited reduced epigenetic aging and improved insulin sensitivity [33]. Plant-based and vegan dietary patterns generally emphasize the consumption of legumes, whole grains (e.g., whole-grain bread, whole-grain cereals, and brown rice), fruits (e.g., blueberries, grapes, and apples), vegetables (including root vegetables and green leafy vegetables), and nuts. These food groups have been associated with a reduced risk of developing diabetes [60].

At the dietary component level, adequate intake of vitamin D (4000 IU/day) with Lactobacillus-containing probiotics (1.8 × 10^9^ CFU/capsule/day), and dietary fiber (15–35 g/day) is recommended, as these interventions have been shown to reduce fasting insulin levels, decrease insulin resistance, and enhance insulin sensitivity [26,36,61]. In contrast, excessive consumption of saturated fats, protein, and fructose should be avoided, as these dietary components promote pro-inflammatory cytokine production and exacerbate insulin resistance [40,41,44].

## 6. Conclusions

Nutrigenomic modulation offers a compelling and preventive approach to type 2 diabetes by targeting insulin signaling and glucose metabolism at the transcriptional and epigenetic levels. The evidence synthesized in this review demonstrates that dietary patterns, caloric intake, and specific nutrients can actively regulate genes involved in insulin sensitivity, inflammation, and energy metabolism, thereby improving metabolic health and reducing diabetes risk. Interventions such as very-low-calorie and plant-based diets, along with targeted supplementation including vitamin D, probiotics, dietary fiber, nucleotides, and bioactive compounds, consistently showed beneficial molecular and metabolic effects, while poor-quality diets rich in fat or fructose promoted proinflammatory and insulin-resistant states. Importantly, gene–diet interactions underscore the need for personalized nutrition strategies to maximize therapeutic efficacy. As diabetes prevalence continues to rise globally, integrating nutrigenomics with precision nutrition and lifestyle modification holds significant potential to shift dietary interventions from supportive measures to core strategies in type 2 diabetes prevention.

## Figures and Tables

**Table 1:** Characteristics of Included Studies.

Study	Study Design	Dietary/Lifestyle Intervention	Target Tissue/Cells	Primary Gene Expression/Molecular Outcomes
Abdelmoneim et al., 2025 [24]	Experimental	Naringenin/naringenin-reduced graphene oxide + high-fat/high-fructose diet	Not applicable	Steatosis, insulin resistance, inflammation
Al-Odinan et al., 2025 [25]	Cross-sectional	Energy, SFA intake	Blood	TCF7L2 rs7903146, insulin, waist circumference
Banikazemi et al., 2025 [26]	RCT	Vitamin D (4000 IU/day), probiotics, combination, placebo (8 weeks)	PBMCs	TNF-α, insulin, hs-CRP
Chen et al., 2025 [27]	Cross-sectional	Lysine, legumes, prebiotics, Mediterranean/prudent diet	Serum	Glyoxylate/dicarboxylate, lysine pathways, GDM
Cheng et al., 2025 [28]	Not applicable	PUFAs (arachidonic acid, DPA)	Orbital, subcutaneous fat	GPR120, PPARγ, lipidomics
Colleluori et al., 2025 [29]	Randomized controlled trial	Diet + aerobic/resistance/combined exercise	Blood, muscle	q, CNTFRα, IGF-1, HOMA-IR, disposition index
Dos Santos et al., 2025 [30]	Cross-sectional + intervention	9-week nutrition program	Blood	GHRL, PLIN1, RETN, NAMPT variants; metabolic markers
Eroglu et al., 2025 [31]	Cohort	Trace elements	Blood	CpG methylation (copper), T2D/CVD risk
Fu et al., 2025 [32]	Cross-sectional	Lifestyle vs. control	Not applicable	Multi-omics: metabolomics, proteomics, methylation
Janssens et al., 2025 [33]	Cohort/cross-sectional	Vegan, vegetarian, pescetarian, omnivore	Blood	DNA methylation, epigenetic age
Jiang et al., 2025 [34]	Observational cohort	Food liking traits	Not applicable	Genetic links: food traits, T2D, CVD
Kumar et al., 2025 [35]	Case–control	4-week very-low-calorie diet (800 kcal/day)	Blood, tissue biopsies	GLUT4 ↓, PDK4 ↑, CPT1 ↑, AMPK ↑
Lima et al., 2025 [36]	Cross-sectional	Dietary fiber	Not applicable	MTNR1B rs10830963, glycemic markers
Maltais-Payette et al., 2025 [37]	Cross-sectional	Not applicable	Liver	GLS1, GLUL, NAGS, amino acids
Moreno-Lopez et al., 2025 [38]	Experimental	SGLT2 inhibitor + diet	Pancreas, islets	FGF21, FGFR1, β-klotho, GSIS
Mostofinejad et al., 2025 [39]	Pilot	High-fat meal	PBMCs	mRNA, miRNA, lncRNA, inflammation, metabolism
Olmedo et al., 2025 [40]	Cross-sectional	Protein, sugars, SFA, food groups	Not applicable	FTO rs9939609, BMI, fat mass, visceral fat, skeletal muscle
Poursharifi et al., 2025 [41]	Experimental (mouse)	High-fat diet	Visceral fat, WAT	ABHD6, PPARs, adiponectin, insulin resistance
Sinke et al., 2025 [42]	Intervention	13-week lifestyle	Muscle, adipose, blood	DNA methylation (>750,000 CpGs), insulin sensitivity genes
Smith & Klein, 2025 [43]	Cross-sectional/observational	Weight loss	Adipose, blood, myotubes	Endotrophin, AKT ser473, insulin signaling
Staltner et al., 2025 [44]	Intervention	Fructose, glucose	Blood monocytes	Toll-like receptor 2 (TLR2), cytokines, specificity protein 1 (SP1)
Tyler et al., 2025 [45]	Experimental (mouse)	Diet-induced obesity	Multi-tissue	Distal transcriptome, metabolic traits
Wagner-Reguero et al., 2025 [46]	Cross-sectional	High sugar/SFA, poor diet	Not applicable	Sweet taste receptor SNPs, metabolic responses
Wang et al., 2025a [47]	Cohort (pre–post)	16-week exercise + diet	Skeletal muscle	505 DEGs (mitochondrial, insulin sensitivity), eQTL/sQTL, metabolic risk genes
Wang et al., 2025b [48]	Observational/mechanistic	Mixed meals, macronutrient loads	Liver, islet, gut	Hormone secretion, multi-omics, insulin resistance prediction
Wang et al., 2025c [49]	RCT	Nucleotide supplement (1.2 g/day, 19 weeks)	Leukocytes	DNA methylation age, HOMA-IR
Wang et al., 2025d [50]	Review	Phytochemicals (curcumin, resveratrol, etc.)	Not applicable	Sirtuin pathway, anti-aging
Wang et al., 2025e [51]	Interventional	Low-calorie diet (815–835 kcal/day, 6 months)	Plasma	Exosomal miRNAs, T2D remission
Zhou et al., 2025 [52]	Experimental (mouse)	Diet-induced obesity	Adipose, liver, muscle	G protein-coupled receptor 65 (GPR65), insulin signaling, inflammation

**Table 2:** Dietary Interventions and Their Effects on Gene Expression Changes and Metabolic Outcomes.

Dietary Component	Gene Expression Changes	Metabolic Effects	Effect Size/Significance
Very-low-calorie diet (800 kcal/day)	↓ GLUT4 (1.57-fold), ↑ PDK4 (3.9-fold), ↑ CPT1 (2.5-fold), ↑ AMPK (2-fold)	↓ body mass index (Δ = 6.21), ↓ glucose (Δ = 6.94), ↓ insulin resistance (Δ = 10.19)	*p* < 0.05 for all
Vitamin D plus probiotics	↓ TNF-α gene activity, ↓ insulin, ↓ insulin resistance, ↑ insulin sensitivity	↓ high-sensitivity C-reactive protein	*p* = 0.007 (TNF-α), *p* = 0.020 (insulin), *p* = 0.024 (insulin resistance)
Dietary fiber (MTNR1B G allele)	↓ Fasting insulin, ↓ HOMA-IR in G allele carriers	Improved glycemic profile	*p* = 0.034 (insulin), *p* = 0.028 (HOMA-IR)
Nucleotides (1.2 g/day)	↓ DNA methylation age	↓ HOMA-IR (β = −0.45)	*p* = 0.0023 (methylation), *p* = 0.033 (HOMA-IR)
Naringenin/naringenin-reduced graphene oxide	Not applicable	↓ Hepatic steatosis, ↓ insulin resistance, ↓ inflammation	Not applicable
Copper (trace element)	↓ CpG site methylation with ↑ serum copper	↓ type 2 diabetes/cardiovascular disease risk with ↑ methylation	Hazard ratio per SD: 0.74–0.52, *p* < 0.05
Polyunsaturated fatty acids (arachidonic acid, docosapentaenoic acid)	↑ GPR120/PPARγ activation	↑ adipose metabolic health	Not applicable
Fructose	↑ TLR2 mRNA, ↑ proinflammatory cytokines	↑ immune response	Not applicable

Upward arrows (↑) denote an increase or activation, whereas downward arrows (↓) denote a decrease or inhibition of the indicated parameters.

**Table 3:** Epigenetic Modifications by Foods and Their Nutrient Derivatives.

Dietary Modifications	Molecular Mechanism	Affected Genes/Pathways	Clinical Relevance
Copper	DNA methylation (CpG)	cg00398673, cg03957124, cg05736499, cg18513344	Increased DNA methylation associated with decreased risk of type 2 diabetes and cardiovascular disease
Vegan/plant-based diet; nucleotides	DNA methylation clocks	Multiple epigenetic clocks (Hannum, Horvath, etc.)	Reduced epigenetic age and improved insulin sensitivity
Low-carbohydrate diet	Exosomal microRNAs	miR-92b-3p, miR-495-3p, miR-452b-5p, PI3K–Akt, FoxO pathways	Prediction of type 2 diabetes remission and modulation of insulin signaling pathways
Fructose	Transcription factor binding	TLR2, SP1	Increased inflammation and immune response

## Data Availability

No new data were created or analyzed in this study. Data sharing is not applicable to this article.
